# Safety and efficacy of hydroxychloroquine for treatment of non-severe COVID-19 among adults in Uganda: a randomized open label phase II clinical trial

**DOI:** 10.1186/s12879-021-06897-9

**Published:** 2021-12-06

**Authors:** Pauline Byakika-Kibwika, Christine Sekaggya-Wiltshire, Jerome Roy Semakula, Jane Nakibuuka, Joseph Musaazi, James Kayima, Cornelius Sendagire, David Meya, Bruce Kirenga, Sarah Nanzigu, Arthur Kwizera, Fred Nakwagala, Ivan Kisuule, Misaki Wayengera, Henry G. Mwebesa, Moses R. Kamya, William Bazeyo

**Affiliations:** 1 Department of Medicine, Makerere University College of Health Sciences, Makerere University, P.O. Box 7072, Kampala, Uganda; 2 Mulago National Referral Hospital, Kampala, Uganda; 3 Infectious Diseases Institute, Makerere University, Kampala, Uganda; 4 Department of Anesthesia, Makerere University, Kampala, Uganda; 5 Makerere University Lung Institute, Kampala, Uganda; 6 Department of Pharmacology, Makerere University, Kampala, Uganda; 7 Department of Microbiology, Makerere University, Kampala, Uganda; 8 Ministry of Health, Kampala, Uganda; 9 School of Public Health, College of Health Sciences, Makerere University, Kampala, Uganda

**Keywords:** COVID-19, Hydroxychloroquine, Outcomes, Treatment, Safety, Efficacy

## Abstract

**Background:**

Several repurposed drugs such as hydroxychloroquine (HCQ) have been investigated for treatment of COVID-19, but none was confirmed to be efficacious. While in vitro studies have demonstrated antiviral properties of HCQ, data from clinical trials were conflicting regarding its benefit for COVID-19 treatment. Drugs that limit viral replication may be beneficial in the earlier course of the disease thus slowing progression to severe and critical illness.

**Design:**

We conducted a randomized open label Phase II clinical trial from October–December 2020.

**Methods:**

Patients diagnosed with COVID-19 using RT-PCR were included in the study if they were 18 years and above and had a diagnosis of COVID-19 made in the last 3 days. Patients were randomized in blocks, to receive either HCQ 400 mg twice a day for the first day followed by 200 mg twice daily for the next 4 days plus standard of care (SOC) treatment or SOC treatment alone. SARS COV-2 viral load (CT values) from RT-PCR testing of samples collected using nasal/orapharyngeal swabs was performed at baseline, day 2, 4, 6, 8 and 10. The primary outcome was median time from randomization to SARS COV-2 viral clearance by day 6.

**Results:**

Of the 105 participants enrolled, 55 were assigned to the intervention group (HCQ plus SOC) and 50 to the control group (SOC only). Baseline characteristics were similar across treatment arms. Viral clearance did not differ by treatment arm, 20 and 19 participants respectively had SARS COV-2 viral load clearance by day 6 with no significant difference, median (IQR) number of days to viral load clearance between the two groups was 4(3–4) vs 4(2–4): p = 0.457. There were no significant differences in secondary outcomes (symptom resolution and adverse events) between the intervention group and the control group. There were no significant differences in specific adverse events such as elevated alkaline phosphatase, prolonged QTc interval on ECG, among patients in the intervention group as compared to the control group.

**Conclusion:**

Our results show that HCQ 400 mg twice a day for the first day followed by 200 mg twice daily for the next 4 days was safe but not associated with reduction in viral clearance or symptom resolution among adults with COVID-19 in Uganda.

*Trial registration: *NCT04860284.

## Background

The novel coronavirus, SARS-CoV-2, which causes Coronavirus disease 2019 (COVID-19), is the seventh human coronavirus described to date. By 3 July 2020, more than 11 million COVID-19 infections were reported worldwide resulting in more than 450,000 deaths [1] In just over a year, there have been nearly 130 million cases with more than two million deaths globally [2]. The COVID-19 pandemic has stretched the health care capacity of all systems across the globe, particularly the low-income countries with the weakest health care systems. Focus has been put on reducing the burden of infection and hospitalization as the primary goal [3]. According to the Uganda Ministry of Health data, the country has had over 41,000 cases and 340 deaths since the first case was reported on 21st March 2020 [4].

Several repurposed drugs have been investigated for treatment of COVID-19, however, none have been confirmed to be efficacious. These drugs include antimalarials like hydroxychloroquine (HCQ), antivirals such as remdesivir and favipiravir and antiretroviral combination therapies such lopinavir/ritonavir. Animal and human studies are not conclusive about the effect of HCQ on covid-19 with one animal study showing no effect, while other in-vitro studies and a small observational study demonstrated antiviral properties of HCQ [5–7]. In Uganda, an observational study among mild COVID-19 patients revealed a shorter time to recovery among those that had received HCQ [8]. Contrary to this, a retrospective study showed slower viral clearance among patients on HCQ compared to standard care [9]. Another randomized open-label trial in mild-to-moderate COVID-19 patients showed no difference in clinical status in the HCQ group as compared to standard of care [8]. However, this trial did not assess viral clearance and included patients up to 14 days after onset of symptoms. The authors asserted that it was conceivable that drugs that may limit viral replication would perhaps be more beneficial in the earlier course of the disease thus slowing progression to severe and critical illness [10]. The contention on the benefit of HCQ remained a debate in Uganda due to conflicting data from higher resource settings.

Despite being ubiquitously used for the treatment of malaria; several studies have highlighted potential harm in the use of HCQ in COVID-19 patients. Cardiac arrhythmias from prolonged QT interval like irregular ventricular rhythms, ventricular tachycardia and fibrillation were noted especially with the relatively high doses administered in some trials to suppress viral replication [11]. However, the populations in these studies were older and burdened with more comorbidities as compared to Uganda’s COVID-19 population.

We therefore performed a randomized, open-label, clinical trial to determine the safety and efficacy measured as viral clearance, of HCQ compared to standard of care (SOC) for treatment of non-severe covid-19 in adults in Uganda.

## Methods

### Study site

We conducted a randomized open label Phase II clinical trial entitled Hydroxychloroquine for Treatment of Non-Severe COVID-19 (HONEST trial) from October–December 2020. The study was conducted at the Namboole nontraditional isolation facility where patients with asymptomatic or mild COVID-19 with no comorbidities were isolated and managed. Namboole stadium, a multipurpose stadium located 10 km east of the central business district of Kampala city, was remodeled into a COVID-19 isolation and treatment facility for patients with asymptomatic and mild COVID-19 due to escalating numbers in the country.

### Study design and population

Diagnosis of COVID-19 was performed using RT-PCR at the government approved laboratories. Patients diagnosed with COVID-19 were included in the study if they were 18 years and above and had a diagnosis of COVID-19 made in the last 3 days. Patients were excluded if they had known allergies to HCQ or chloroquine, were on medications that have clinically significant interactions with HCQ, had a positive rapid test for malaria, were diagnosed with severe/critically ill COVID-19 (WHO Ordinal Scale of ≥ 5), had QTc prolongation of > 450 ms for males and > 470 ms for females, were pregnant or breastfeeding or were on chronic HCQ use. Participants found to have hypo- or hyperkalemia at baseline were withdrawn from the study.

### Randomization and masking

Randomization was performed by an independent statistician using a computer generated randomization code with block randomization with varied sizes. Patients were allocated in a ratio of 1:1; to receive either HCQ 400 mg twice a day for the first day followed by 200 mg twice daily for the next 4 days plus SOC treatment or SOC treatment alone. The SOC treatment at the time included vitamin C and zinc supplementation. Symptomatic patients also received azithromycin and analgesics if necessary. Computer-generated randomization codes were enclosed in sequentially numbered opaque sealed envelopes containing treatment allocation. After meeting study eligibility criteria, the study nurse assigned the next envelope to the participant, opened the envelope and assigned treatment allocation. Treatment was immediately initiated. Participants and the trial team were not blinded. Participants who progressed to WHO ordinal scale ≥ 5 (severe or critical disease) during the study were managed according to the national clinical guidelines for COVID-19 which includes intravenous antibiotics and anticoagulation with low molecular weight heparin.

### Clinical assessments

Participants were evaluated daily for new clinical symptoms, worsening or improvement of existing symptoms and adverse events during admission. An ECG was obtained at baseline, day 2 and 4. Where the QTc interval on ECG exceeded 500 ms or increased by > 60 ms above the baseline, the ECG was repeated. If the repeat QTc interval remained above these values, HCQ was discontinued. Serum ALT, visual tests using Snellen’s and Ishihara charts were measured at baseline and day 4 while serum potassium was measured only at baseline. Participants who developed grade 3 or 4 clinical or laboratory based adverse events were discontinued from medication. Participants were discharged after 2 consecutive negative SARS COV-2 PCR tests, generally after 10–14 days.

### Virological assessments

SARS COV-2 viral load (CT values) from RT-PCR testing of samples collected using nasal/orapharyngeal swabs was performed at baseline, day 2, 4, 6, 8 and 10. Following nasal/oropharyngeal swabbing, samples were stored in sterile saline solution and transported in a cooler box to the Infectious Diseases Institute Core Laboratory, which is a government approved laboratory for SARS COV2 PCR testing.

### Ethical considerations

Ethics approval was obtained from the Makerere University School of Medicine Research and Ethics Committee (#REC REF 2020-137), the Uganda National Council for Science and Technology (RESCLEAR/05) and the National Drug Authority (CTA 0143). Written informed consent was obtained from all study participants and the trial was conducted according to Good Clinical Practice Guidelines in accordance with the Declaration of Helsinki. The trial was registered with ClinicalTrials.gov on 26/04/2021 (registration number NCT04860284).

### Interim analyses

An independent data and safety monitoring board (DSMB) reviewed the study protocol and oversaw the progress of the trial. Progressive data review for safety and efficacy was planned after 25% of the participants had completed 10 days of follow-up, and another as deemed necessary by the DSMB. Stopping guidelines were provided to the DSMB with the use of a Lan–DeMets spending function for the primary outcome. The first interim analysis was performed and presented to the DSMB committee on February 17, 2021, for their recommendation on whether to stop the trial for safety concern or futility or any other reason given by the committee. The trial stopped because of the national roll-out of HCQ as standard of care by the Uganda Ministry of Health.

### Data analysis

Primary and secondary outcomes were analyzed on intention-to-treat population, and other outcomes and safety data were analyzed on complete cases. The primary outcome was median time from randomization to SARS COV-2 viral clearance by day 6. Viral load clearance was defined as a negative SARS COV-2 PCR test with no subsequent positives. Analysis of time to viral clearance was performed using Kaplan–Meier methods, and compared across the two treatment arms using log-rank test. We used Cox regression model to compare the secondary outcome of rates of viral load clearance in the two arms. Proportional hazard assumption was checked using schonefeld residuals. For other outcomes: the proportion of PCR negative conversion by day 6 and day 10, and proportion of participants with 25% reduction of SARS COV-2 viral load (CT-values) from baseline at day 6 were compared between treatment arms using Chi square test; change in SARS COV-2 viral load (CT-values) over time in the two arms compared using student’s T-test; Time to symptom clearance by day 10 was summarized using median and inter-quartile range and compared using Wilcoxon rank-sum test. Safety outcomes like incident elevated ALT (> 40 IU), incident elevated QTc interval (QTc > 450 ms in males and QTc > 470 ms in females), incident color vision loss/deficiency and adverse events were summarized using frequencies and percentage, across the treatment arms. We conducted all analyses with STATA software, version 15.1 (Texas, USA), according to the intention-to-treat principle, with two-sided type I error of 5%. A participant who did not achieve viral clearance or was lost to follow-up, withdrawn or died before analysis time (day 6 or day 10) or had missing SARS COV-2 viral load and PCR test results, was assigned non-viral load clearance at their time of censoring. Sensitivity analyses of viral clearance were performed using an adjusted Cox regression model, specified a priori included baseline age and sex. Baseline CT values and body mass index (BMI) were not included in the adjusted regression model due to very high missing values. Baseline SARS COV-2 CT-values were measured at patient enrolment. However, majority of the patients were enrolled at day 4 after first positive PCR tests. There was no correction for multiplicity on tests for secondary/other outcomes so results are reported as point estimates and 95% confidence intervals. Confidence interval widths are not adjusted for multiplicity, so intervals should be interpreted with caution.

We assumed HCQ would lower median time to viral clearance from 7 days (as per standard of care) to 4 days, with power of 80% and 5% 2-sided significance level, we calculated a sample size of 284 patients (142 per group) after accounting for 25% loss to follow-up or missing data.

## Results

**Fig. 1:**
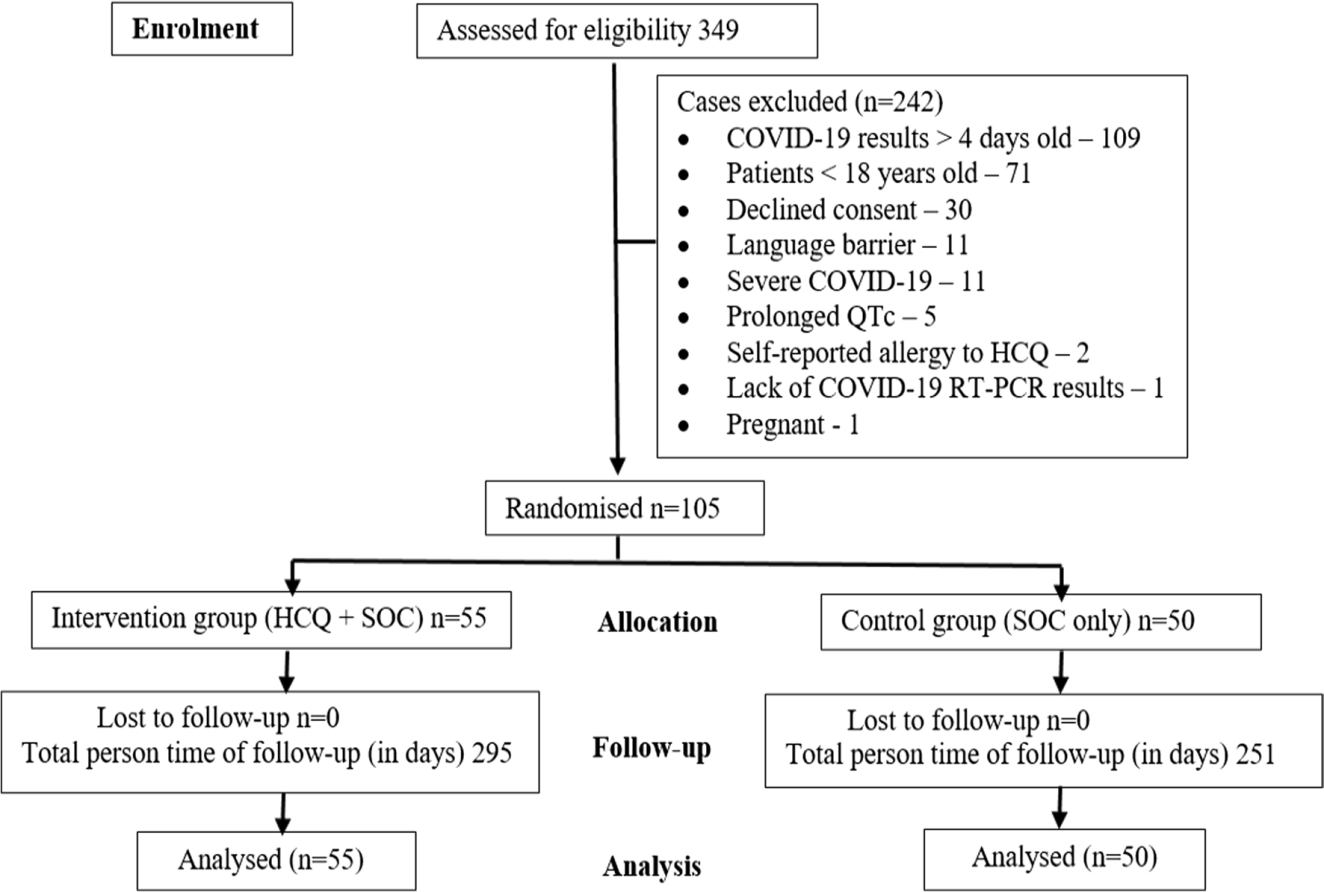
Disposition of the study participants

Of the 105 participants enrolled, 55 were assigned to the intervention group (HCQ plus SOC) and 50 to the control group (SOC only). The proportion of target (284) enrolled was 37%. Figure 1 shows the disposition of the study participants.

Table 1 shows the baseline characteristics of the participants. The median (IQR) age was 32 (27–43) years and majority 76 (72.4) were male. Regarding COVID-19 symptoms at baseline, cough was the most common in 24 (43.6%) participants in the intervention group and 21 (42.9%) in the control group, followed by headache 14 (25.5%) and 11 (22.4%) in intervention and control group respectively. Details of the baseline COVID-19 symptoms are as shown in Table 2.

**Table 1:** Baseline socio-demographics of participants

Characteristics	Arm 1: HCQ + SOC	Arm 2: SOC alone	Total
Total randomized and analyzed	55	50	105
*Age in years*			
Median (IQR)	30 (26–44)	32 (27–42)	32 (27–43)
Range	18–64	20–59	18–64
Age categories, n (%)			
18–34	29 (52.7)	28 (56.0)	57 (54.3)
35–59	25 (45.5)	22 (44.0)	47 (44.8)
≥ 60	1 (1.8)	0	1 (0.9)
*Sex, n (%)*			
Male	39 (70.9)	37 (74.0)	76 (72.4)
Female	16 (29.1)	13 (26.0)	29 (27.6)
*Baseline SARS COV-2 CT-values*^a^			
Number of observations	17	17	34
Mean (SD)	19.0 (5.1)	18.9 (4.9)	18.9 (4.9)
Median (IQR)	18.8 (16.1–23.0)	19.9 (14.8–22.4)	19.6 (14.8–23.0)
Range (minimum–maximum)	9.6–27.1	9.6–25.5	9.6–27.1
*Comorbidity, n (%)*			
High blood pressure^b^	2 (3.6)	1 (2.1)	3 (2.9)
Heart disease	1 (1.8)	0	1 (0.9)
Diabetes^b^	2 (3.5)	1 (2.0)	3 (2.8)
Cigarette smoking^b^	2 (3.6)	0	2 (1.9)
Alcohol dependency^b^	7 (12.3)	6 (12.5)	13 (12.4)
HIV positive^b^	3 (5.4)	2 (4.1)	5 (4.8)

*HCQ* hydroxychloroquine, *SOC* standard of care, *SD* standard deviation, *IQR* Inter-quartile range

^a^The baseline SARS COV-2 CT-values were defined as the CT-values measured at patient’s enrolment. However, some participants had missing CT values at enrolment because majority of participants reported 4 days after their first positive PCR tests, and the repeat PCR test at enrolment for most of them was negative, as highlighted in this table. Only positive PCR tests could have SARS COV-2 CT-values

^b^Missing values: High blood pressure (n = 2), Heart disease (n = 1), Diabetes (n = 1), Cigarette smoking (n = 2), Alcohol dependency (n = 2), HIV status (n = 1), History of allergies (n = 1), Medication before admission (n = 1)

**Table 2:** Baseline COIVD-19 symptoms

Characteristics	Arm 1: HCQ + SOC	Arm 2: SOC alone	Total
Number randomized	55	50	105
Number with baseline symptom information^a^	55	49	104
*General symptoms, n (%)*			
Fever	5 (9.1)	4 (8.2)	9 (8.7)
Tiredness	5 (9.1)	3 (6.1)	8 (7.7)
Muscle aches	4 (7.3)	5 (10.2)	9 (8.7)
*Cardio-respiratory, n (%)*			
Cough^b^	24 (43.6)	21 (42.9)	45 (43.3)
Running nose	11 (20.0)	4 (8.2)	15 (14.4)
Nasal congestion	5 (9.1)	3 (6.1)	8 (7.7)
Sore throat	3 (5.5)	2 (4.1)	5 (4.8)
Difficulty in breathing	1 (1.8)	0	1 (0.9)
Fast breathing	0	0	0
Chest pain	7 (12.7)	5 (10.2)	12 (11.5)
*Neurological, n (%)*			
Headache	14 (25.5)	11 (22.4)	25 (24.0)
Dizziness	1 (1.8)	3 (6.1)	4 (3.8)
Loss of smell	7 (12.7)	5 (10.2)	12 (11.5)
Loss of taste	5 (9.1)	4 (8.2)	9 (8.7)
*Gastrointestinal, n (%)*			
Poor appetite	5 (9.1)	4 (8.2)	9 (8.7)

^a^1 participant (in SOC alone) had missing baseline symptom information. Percentages computed on complete cases

^b^73% (63% in HCQ group, 86% in SOC group) had dry cough among those reported cough

The proportions of participants’ clinical and laboratory examination features at baseline did not differ between groups (Table 3).

**Table 3:** Baseline clinical and laboratory features

Characteristics	Arm 1: HCQ + SOC	Arm 2: SOC alone	Total
Number randomized	55	50	105
Body mass index (BMI) in kgs/m^2 a^			
Number of observations	55	49	104
Mean (standard deviation)	30.8 (7.5)	31.3 (7.3)	31.0 (7.4)
BMI categories			
< 18.5	1 (1.8)	1 (2.0)	2 (1.9)
18.5 to < 25 (normal)	13 (23.6)	9 (18.4)	22 (21.2)
25 to < 30 (over-weight)	14 (25.5)	12 (24.5)	26 (25.0)
≥ 30 (obese)	27 (49.1)	27 (55.1)	54 (51.9)
ECG QTc interval (ms)			
Overall–mean (SD)	417 (22)	409 (20)	413 (22)
Males–mean(SD)	410 (22)	404 (19)	407 (21)
Females–mean(SD)	430 (15)	423 (16)	427 (16)
Serum Potassium (mmols/L)			
Number of observations	52	41	93
Mean (SD)	4.6 (0.9)	4.6 (0.7)	4.6 (0.8)
Visual color test, (%)			
Normal	54 (94.7)	50 (100.0)	104 (97.2)
Abnormal	3 (5.3)	0	3 (2.8)
Pulse rate (beats/min), median (IQR)	78 (70–86)	78 (67–85)	78 (68–86)
Visual acuity–left eye, n (%)			
6/6	42 (76.4)	37 (77.1)	79 (76.7)
5/6	4 (7.3)	1 (2.1)	5 (4.9)
4/6	2 (3.6)	0	2 (1.9)
20/30	6 (10.9)	8 (16.7)	14 (13.6)
6/12	1 (1.8)	1 (2.1)	2 (1.9)
6/18	0	1 (2.1)	1 (1.0)
Visual acuity–right eye, n (%)			
6/6	43 (75.4)	38 (82.6)	81 (78.6)
5/6	5 (8.8)	0	5 4.9)
4/6	2 (3.5)	1 (2.2)	3 (2.9)
6/12	0	1 (2.2)	1 (1.0)
20/30	7 (12.3)	6 (13.0)	13 (12.6)

^a^Missing values: BMI, 1 (0.95%)

Of 55 participants in the intervention group and 50 in the control group, 20 and 19 participants respectively had SARS COV-2 viral load clearance by day 6 with no significant difference, median (IQR) to viral load clearance between the two groups was 4(3–4) vs 4(2–4) days: p = 0.457 as shown in Table 4.

**Table 4:** Comparison of primary and secondary outcomes

Outcomes	Arm 1: HCQ + SOC	Arm 2: SOC alone	P-value
Number randomized and analyzed (intention-to-treat population)^a^	55	50	105
Median (IQR) Time (in days) to SARS COV-2 viral load clearance by day 6^b,c^	4 (3–4)	4 (2–4)	0.457
Total person-time of follow-up (in days)	295	251	N/A
Number of patients with viral load clearance by day 6	20	19	N/A
Rate of viral load clearance per 100 person-days (95%CI)	6.8 (4.4–10.5)	7.6 (4.8–11.7)	N/A
Sensitivity analysis (on viral clearance) adjusted analysis—Hazard Ratio (95% CI)^d^	0.84 (0.44–1.61)		0.607
Proportion PCR negative conversion by day 6, n(%)	20 (35.1)	19 (38.0)	0.755
Proportion PCR negative conversion by day 10, n(%)	28 (49.1)	27 (54.0)	0.615
Number of patients with CT values data at both baseline and follow-up^e^	15	15	N/A
Change in CT-values from baseline, mean (SD)	5.8 (5.3)	4.1 (7.1)	0.471
Proportion with 50% reduction of SARS COV-2 viral load (CT-values) from baseline at day 6, n(%)	5 (33.3)	6 (40.0)	0.705
Proportion with 25% reduction of SARS COV-2 viral load (CT-values) from baseline at day 6, n(%)	8 (53.3)	6 (40.0)	0.464
Median (IQR) time in days to symptom clearance by day 10^f^	3 (2–5)	3 (2– 5)	0.909
*Laboratory safety outcomes*			
Number of patients with ALT data at day 0 and 4	46	38	N/A
Incident ALT > 40 IU at day4, n (%)	4 (8.7)	5 (13.2)	N/A
Incident elevated QTc interval			
Male–number of participants	39	37	N/A
Incident elevated QTc > 450 ms	1 (2.6)	3 (8.1)	
Female–number of participants	16	13	N/A
Incident elevated QTc > 470 ms	1 (6.3)	0	
Incident color vision loss/deficiency at day 4	0	0	N/A

*PCR* polymerase chain reaction, *CT values* cycle threshold values

^a^Intention-to-treat analysis but with early stopping at an estimated 37% (105) of required sample size of 284

^b^A participant was considered as having attained PCR negative conversion at first negative SARS COV-2 PCR test but without subsequent positive PCR test

^c^Time to viral load clearance was estimated in only those who had viral load clearance. Majority of patients were enrolled at day4 after first positive PCR tests. Only 20/104 (19.2%) had day 2 PCR test results

^d^Cox proportional hazard regression model adjusting for age groups () and gender

^e^CT-values data is analyzed for only positive PCR tests at day 6, therefore, we expect less numbers here

^f^Time to symptom clearance was estimated in only participants who reported a symptom at baseline

**Fig. 2:**
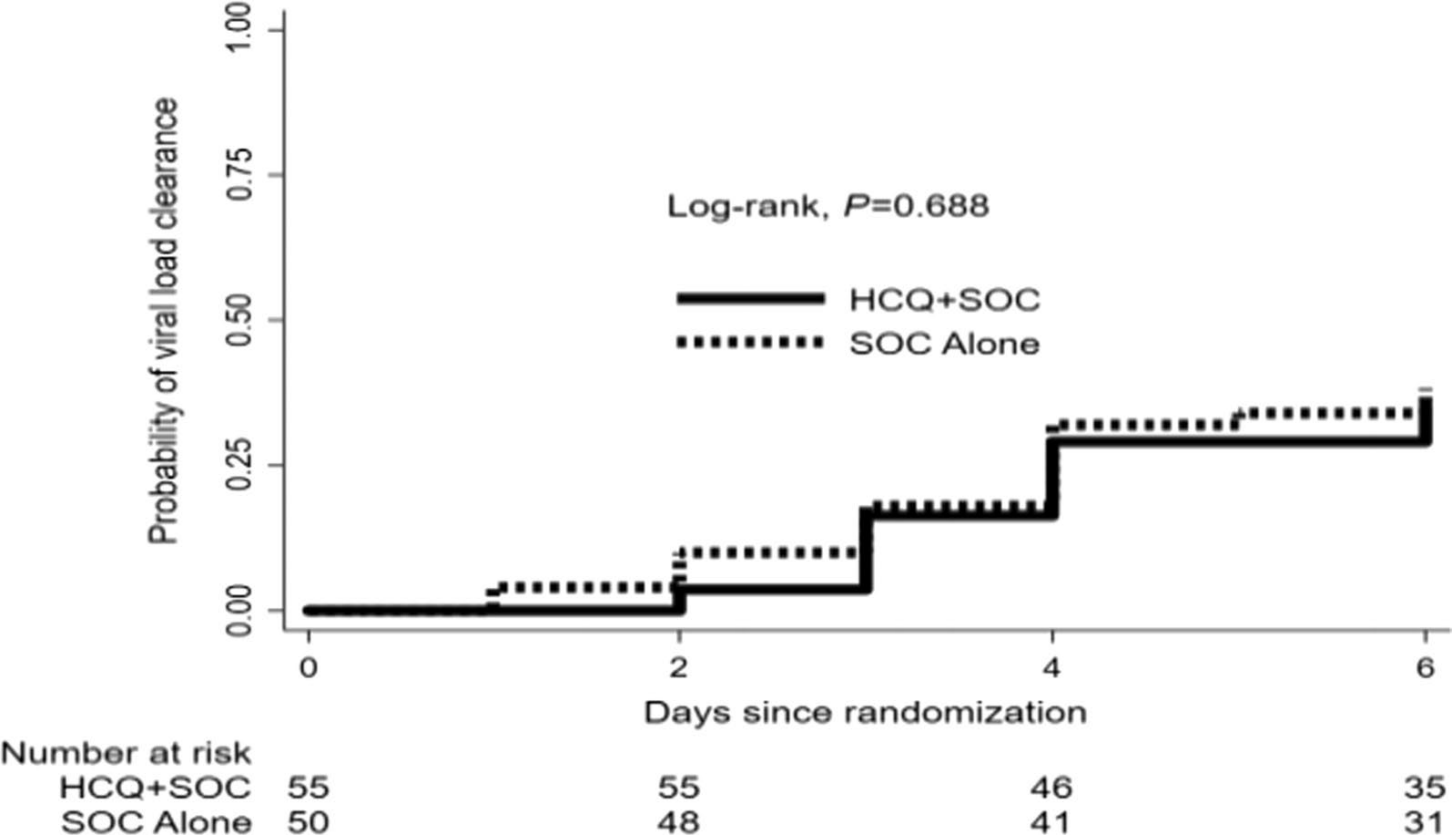
Kaplan–Meier plot showing time to first SARS COV-2 viral load clearance by treatment groups

Figure 2 shows the Kaplan–Meier plot showing time to first SARS COV-2 viral load clearance by treatment groups. The rate of viral load clearance per 100 person-days (95% CI) did not differ between the intervention and control groups, unadjusted hazard ratio 0.89 (95% CI 0.47–1.66): p = 0.703 (Table 4).

There were no significant differences in secondary outcomes between the intervention group and the control group as shown in Table 4: SARS COV-2 PCR negative conversion by day 6 was found in 20 (35.1%) participants in the intervention group vs 19 (38.0%) participants in the control group, p = 0.755. Of 55 participants in the intervention group and 50 in the control group, SARS COV-2 CT values data were available for 15 participants in each group. There was no significant difference in change in CT values from baseline (mean, SD) in the intervention group 5.8 (5.3) vs 4.1 (7.1) in the control group, p = 0.471. The proportion with 50% reduction of SARS COV-2 viral load (CT values) from baseline was not statistically significant in the intervention group 5 (33.3%) vs 6 (40.0) in the control group, p = 0.464, by day 6.

Regarding COVID-19 symptoms, data were available for 36 participants in the intervention group and 29 in the control group. There was no significant difference in time to symptom clearance by day 10 between the two groups (median (IQR) in days 3 (2–5) vs 3 (2–5): p = 0.909), this finding was similar to individual symptom analysis (Table 5).

**Table 5:** Time to symptom clearance for individual symptoms by day 10

	Arm 1: HCQ + SOC		Arm 2: SOC	
	No. patients	Median (IQR) days	No. patients	Median (IQR) days
Overall	36	3 (2–5)	29	3 (2–4)
General symptoms				
Fever	5	1 (1–2)	4	1 (1–3)
Tiredness	5	1 (1–1)	3	1 (1–2)
Muscle aches	4	2 (1–3)	5	1 (1–1)
Cardio-respiratory symptoms				
Cough	20	3 (2–4)	14	4 (2–5)
Running nose	11	1 (1–3)	4	2 (1–3)
Nasal congestion	4	4 (3–5)	3	2 (1–5)
Sore throat	3	1 (1–3)	2	1 (1–1)
Difficulty in breathing	1	1 (1–1)	0	0
Chest pain	7	1 (1–4)	5	2 (1–3)
Neurological symptoms				
Headache	14	2 (1–3)	11	2 (1–5)
Dizziness	1	1 (1–1)	3	2 (1–4)
Loss of smell	6	2 (1–3)	4	1 (1–3)
Loss of taste	4	1 (1–2)	4	1 (1–1)
Gastrointestinal				
Poor appetite	5	3 (1–5)	4	1 (1–2)
Nausea	1	1 (1–1)	0	N/A
Vomiting	1	1 (1–1)	0	N/A
Abdominal pain	4	1 (1–3)	2	2 (1–3)
Diarrhea	1	1 (1–1)	1	5 (5–5)

### Safety of HCQ

There were no significant differences in adverse events such as elevated alkaline phosphatase and prolonged QTc interval among patients in the intervention and control group. Details of clinically significant laboratory abnormalities are as shown in Table 6.

**Table 6:** Adverse events

Events	Arm 1: HCQ + SOC	Arm 2: SOC
Number randomized	55	50
Participants who had any adverse event (AE), n (%)^a^	33 (60)	27 (54.0)
Participants who had any severe adverse event (SAE), n (%)^a^	0	0
Participants who had any grade 3 or 4 adverse events, n (%)^b^	2 (6.1)	3 (11.1)
Number of grade 3 or 4 AEs	2	3
Grade 3 or 4 adverse events listing, number		
Elevated QTc (> 450 males, > 470 females)	1	3
Painful eye after HCQ dosages	1	0
Grade 3 or 4 relationship with study drug, number		
Definite^c^	1	N/A
Probable	0	N/A
Possible	0	N/A
Unlikely	0	N/A
Unrelated	1	N/A

^a^Percent of total participants randomized

^b^Percent of those who had any AE

^c^Definite related to study drugs was elevated QTc

## Discussion

In this randomized, open-label, clinical trial to determine the safety and efficacy, of HCQ for treatment of non-severe SARS CoV-2 PCR-positive adults in Uganda, we found no difference in the proportion of participants who had PCR negative conversion, a 50% reduction in SARS-CoV-2 viral load (based on Ct values) after 6 days of treatment, or resolution of symptoms by day 10 of treatment when we compared participants who were randomized to receive HCQ and participants receiving SOC.

Since March 2020, various therapies have been evaluated in clinical trials with adoption of some in clinical guidelines. One of these therapies, HCQ, was first used in 1955 and is considered to have a superior safety profile over chloroquine [12]. In vitro studies suggest that HCQ prevents SARS-CoV-2 binding to gangliosides, subsequently preventing binding with the Angiotensin-converting enzyme receptor (ACE-2), required for viral entry into cells [13]. By incorporating into endosomes and lysosomes, the drug increases the pH of intracellular compartments, resulting in defective protein degradation, endocytosis, and exocytosis required for viral infection, replication, and propagation [14]. HCQ was shown to inhibit a broad range of viruses including coronaviruses (SARS-CoV-1 and Middle East respiratory syndrome-CoV) in cell culture [15, 16], however, evidence from Hamster models suggested that HCQ did not demonstrate an effect on reducing SARS-CoV-2 virus levels [17].

By 13 April 2021, 62 trials of HCQ for the treatment of COVID-19 had been completed [18]. The efficacy of HCQ has been explored in both mild-moderate and severe COVID-19 disease. Similar to our study, Chen and colleagues did not find statistically significant differences in PCR conversion rate by day 7 and no difference was observed in clinical outcomes [19]. The SOLIDARITY trial conducted in multiple countries did not demonstrate mortality benefit among hospitalized patients who were treated with HCQ [20]. Omrani et also found HCQ to be safe with no sever adverse events when used with or without azithromycin, however it had no effect on virological outcomes at day 14 [21]. In a trial evaluating the efficacy of HCQ and standard of care vs standard of care alone, Tang and colleagues showed that the addition of HCQ did not result in a significantly higher probability of negative PCR conversion by 28 days [22]. In outpatient settings, HCQ has also shown mixed efficacy when used as post-exposure prophylaxis with one study in India showing a relative reduction in the incidence of COVID-19 [23] while two other trials in the United States and Canada and one recent metanalysis did not demonstrate any benefit in prevention of COVID-19 [24–26]. Further, the use of once or twice weekly or daily (over 8 weeks) HCQ as pre-exposure prophylaxis among health care workers did not significantly reduce the incidence of laboratory confirmed SARS-CoV-2 infection [27, 28].

Two metanalysis, one of which was conducted more recently, showed that there was insufficient evidence to demonstrate the efficacy of HCQ in reducing short term mortality or risk of hospitalization among outpatients with SARS-CoV-2 infection [29, 30]. One study combining HCQ with azithromycin demonstrated significantly reduced viral titers at day 6 resulting in shortened time to clinical recovery and cough remission [6], however, the sample size was small, and the severity of disease was not clearly stated. Although the U.S. Food and Drug Administration had issued Emergency Use Authorization for the use of HCQ to treat COVID-19 in adolescents and adults on 28 March 2020 [31], this authorization was later revoked on April 15, 2020 due to growing evidence of cardiac adverse events along with evidence suggesting that the drug was unlikely to be effective in treating COVID-19 [32].

Additionally, in March 2021 a WHO expert panel review of studies testing HCQ for preventing COVID-19, found high certainty evidence indicating HCQ has no significant impact on mortality risk or hospitalization and also found moderate certainty evidence that the drug does not significantly impact the risk of developing COVID-19.

The most common side effects of HCQ include nausea, vomiting, and diarrhea [33], however, prolongation of the QTc interval has been observed with HCQ use and can result in ventricular arrythmias [12]. In a Spanish trial involving asymptomatic contacts of patients with polymerase-chain-reaction (PCR)–confirmed COVID-19, the incidence of adverse events was higher in the HCQ group than in the usual-care group (56.1% vs. 5.9%), but no treatment-related serious adverse events were reported [34]. We found no excess occurrence of these adverse events in the arm using HCQ compared to the SOC arm during our trial.

In December 2020, the Uganda Ministry of Health adopted the use of HCQ for treatment of mild to moderate COVID-19 disease, subsequently, on 8 February 2021, the Uganda National Council of Science & Technology issued a directive halting this trial after enrollment of 37% (105) of estimated sample size of 284 participants. Thus, the trial did not reach the planned sample size. There was slower recruitment of participants when management of patients with asymptomatic disease was changed to include home based self-isolation and treatment from the previous recommendation of hospitalizing all those with a positive SARS-CoV-2 test. Despite this limitation, our study was still able to provide locally generated evidence to add to the body of evidence regarding the study question.

In conclusion, our results show that HCQ 400 mg twice a day for the first day followed by 200 mg twice daily for the next 4 days was safe but not associated with reduction in viral clearance or symptom resolution among adults with COVID-19 in Uganda. These findings do not support the use of HCQ in the management of non-severe COVID-19 disease and we recommend the exclusion of HCQ from Ugandan COVID-19 treatment guidelines.

## Data Availability

The data-sets used and analyzed during the current study are available from the corresponding author upon reasonable request.

## Abbreviations

HCQ: Hydroxychloroquine
COVID-19: Corona virus disease 2019
RT-PCR: Real time polymerase chain reaction
SOC: Standard of care
HONEST trial: Hydroxychloroquine for Treatment of Non-Severe COVID-19
CT-values: Cycle threshold values
SARS COV-2: Severe acute respiratory syndrome corona virus 2
